# Epigallocatechin Gallate for Management of Heavy Metal-Induced Oxidative Stress: Mechanisms of Action, Efficacy, and Concerns

**DOI:** 10.3390/ijms22084027

**Published:** 2021-04-14

**Authors:** Iwona Zwolak

**Affiliations:** Centre for Interdisciplinary Research, Laboratory of Oxidative Stress, The John Paul II Catholic University of Lublin, Konstantynów Ave. 1J, 20-708 Lublin, Poland; iwona.zwolak@kul.pl

**Keywords:** epigallocatechin gallate, EGCG, antioxidant, heavy metals, reactive oxygen species, oxidative stress

## Abstract

In this review, we highlight the effects of epigallocatechin gallate (EGCG) against toxicities induced by heavy metals (HMs). This most active green tea polyphenol was demonstrated to reduce HM toxicity in such cells and tissues as testis, liver, kidney, and neural cells. Several protective mechanisms that seem to play a pivotal role in EGCG-induced effects, including reactive oxygen species scavenging, HM chelation, activation of nuclear factor erythroid 2-related factor 2 (Nrf2), anti-inflammatory effects, and protection of mitochondria, are described. However, some studies, especially in vitro experiments, reported potentiation of harmful HM actions in the presence of EGCG. The adverse impact of EGCG on HM toxicity may be explained by such events as autooxidation of EGCG, EGCG-mediated iron (Fe^3+^) reduction, depletion of intracellular glutathione (GSH) levels, and disruption of mitochondrial functions. Furthermore, challenges hampering the potential EGCG application related to its low bioavailability and proper dosing are also discussed. Overall, in this review, we point out insights into mechanisms that might account for both the beneficial and adverse effects of EGCG in HM poisoning, which may have a bearing on the design of new therapeutics for HM intoxication therapy.

## 1. Background

Heavy metal (HM) pollution is one of the major public health concerns in Europe and beyond. Features of heavy metals that make them particularly dangerous for humans include their high toxicity potential, the ability to bioaccumulate in plants, and the biological buildup in the food chain [1,2]. In addition, HMs as pollutants are not degradable and can persist in the environment for a long time, thereby posing a significant threat to human health [1]. For example, a high concentration of arsenic (As) is still being reported in the topsoil of the historical mining areas in Southern Saxony (Germany) [3], and a high mercury (Hg) concentration has been found in the soils of the former mining Middle Spiš area in Slovakia [4]. Sodango et al. [5] reported that as much as 10.18% of farmland soils, which support 13.86% of grain production in China, are affected by HM pollution originating mainly from anthropogenic activities. Other data report that HM contamination may significantly contribute to the cancer epidemic in sub-Saharan Africa [6]. Clearly, people working in an industry that uses HMs such as mining industry [7], automotive parts industry [8], nonferrous metal industry [9], or tile industry [10] are at higher risk of exposure to HMs. However, others may also be threatened by potential HM exposure, for example through living in HM-polluted industrial areas [11] or prolonged ingestion of HM-contaminated cereals or fish [12] (more examples of non-occupational sources of HM exposure are shown in Table 1). The continuing danger from HM exposure is supported by the fact that as many as four HMs (As, Pb, Hg, and Cd) have been included in the top 10 in the Agency for Toxic Substances and Disease Registry (ATSDR) priority list of hazardous substances [13]. This list identifies substances that ATSDR and the Environmental Protection Agency (EPA) consider as “the most significant potential threat to human health due to their known or suspected toxicity and potential for human exposure” at Superfund sites in the U.S. [13]. In addition, four HMs (i.e., As, Cd, Cr(VI), and Ni) have been classified as class 1 carcinogens by the International Agency for Research on Cancer [14], which implies that they are known to cause cancer in humans. Furthermore, HM toxicity has been linked with neurological disorders, kidney dysfunction, allergy, and male and female infertility [15,16].

Chelation therapy is the major pharmacological method used for the treatment of toxic metal poisoning [28]. In this therapy, a chelating agent combines with metal ions forming a stable ring-like structure called a chelate. The chelate structure is more water-soluble than the toxic metal, which facilitates removal of the toxic metal from tissues and excretion thereof by kidneys [29,30]. However, there are drawbacks associated with chelation therapy, mainly with regard to the potentially adverse health effects of chelators, such as gastrointestinal symptoms, nephrotoxicity, and neurological effects [28]. There are also other concerns. For example, dimercaprol (BAL) is often dissolved in peanut oil, which may induce allergic reaction in people susceptible to peanuts. Moreover, this chelating agent has a narrow therapeutic window and is administered through deep intramuscular painful injections [28,31]. BAL and CaNa_2_EDTA (calcium disodium ethylenediamine tetraacetic acid) were reported to redistribute As and Pb, respectively, from other tissues to the brain, and DMSA (meso-2,3-dimercaptosuccinic acid) was shown to increase Hg levels in motor axons [29]. In addition, the efficiency of chelating agents is not always satisfactory. For example, as reported by Li et al. [32], treatment of As poisoning with chelating agents brings positive short-term effects, whereas the long-term effects of treatment with chelators are below expectations. Currently, no effective chelating agents can be recommended for the treatment of Cd or Cr toxicity in humans [28]. Lastly, the high cost of chelation treatment hinders its use in developing countries such as sub-Saharan countries where HM pollution is regarded as a significant health risk to the population [6,15]. Therefore, there is a need for development of potential alternative methods with antidotes that will help to increase the efficiency of chelators or allow substituting these compounds.

Although the efficacy of chelation therapy in the treatment of HM toxicity is undebatable, new approaches are being developed to enhance clinical treatment of HM toxicity and reduce potential adverse effects that may arise from the chelation treatment. One of such approaches includes combining chelation therapy with administration of antioxidants or the use of antioxidants alone. Such combination therapy (chelator and antioxidant) has been suggested to increase toxic metal mobilization and excretion and reduce oxidative stress markers [29,31]. Different natural antioxidant-rich food products have been proposed to be included in a daily diet as a safe and cheap way of blocking the adverse effects of HMs on the human body [20,33,34]. They include edible plants like garlic (*Alium sativum*), onion, broccoli, coriander, *Ginkgo biloba*, and green tea (*Camellia sinensis*) [15,20,35]. Among them, green tea receives significant attention in this regard. Green tea has been reported to prevent As-induced neurotoxicity in rats [36], reduce the bioaccessibility of dietary mercury [37], and protect against Cd-induced damage to rat testes [38]. Green tea solutions have been found to prevent Pb and Cd toxicity in animals through enhancement of endogenous antioxidant mechanisms and chelation of these metals (reviewed by Winiarska-Mieczan [33]). The polyphenol epigallocatechin gallate (EGCG), belonging to the chemical class of flavan-3-ols (or catechins), stands out as the most active and well-studied antioxidant in green tea.

**Figure 1:**
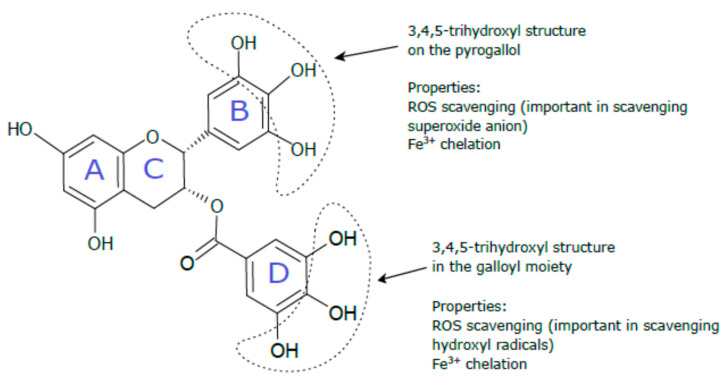
Epigallocatechin gallate (EGCG) structure. EGCG is composed of four rings designated as A, B, C, and D. The A and C rings form a benzopyran ring system, which is connected with the pyrogallol (the B ring) and gallate (the D ring) moiety at the C-2 and C-3 positions, respectively [45]. The role of the functional groups in the antioxidant properties of EGCG was described on the basis of other studies [49,50,51].

EGCG (Figure 1) is the main green tea catechin accounting for 50–80% of the total catechin content [39]. EGCG is characterized by its antioxidant capacity through scavenging free radicals species or chelating metal ions [40]. Some studies show that EGCG is the most powerful catechin in scavenging intracellular ROS [41,42] and a more efficient ROS scavenger than vitamin C [43]. EGCG has also been demonstrated to have anti-inflammatory and anticancer properties [44,45]. There is a wealth of literature describing the beneficial effects of EGCG on various disorders, e.g., metabolic syndrome [40], kidney diseases [46], neurological diseases [47], and cancer [48]. The suggested beneficial properties of EGCG, including the antioxidant, anti-inflammatory, and anticancer effects, are very desirable features for its application in the prevention of HM toxicity. Therefore, unsurprisingly, many researchers have challenged this compound in the therapy of HM poisoning.

The EGCG-based treatment against HMs has been studied for more than a decade, but no comprehensive review and no extensive analysis have been conducted so far. To this end, we collected all studies published up to 2021 examining the effectiveness of EGCG treatment in HM intoxication in animal and cell culture models. In this review, we concentrate on studies that used pure EGCG (studies with GT extracts or GT infusions were excluded) to put the EGCG-specific actions in the center of attention. First, we shortly describe the current health risks related to each of the HMs (i.e., lead, arsenic, cadmium, chromium (VI), nickel, and mercury) in order to give a more updated picture of these metals to the reader. Secondly, based on the collected studies, we delineate EGCG-induced protective mechanisms against these HMs involving free radical scavenging, direct interaction of EGCG with HMs and enhancement of their excretion, enhancement of antioxidant defenses, and anti-inflammatory effects. In addition, since mitochondrial ROS generation is an important step in HM-induced cell damage [52], we also discuss the possible role of these organelles in the beneficial effects of EGCG. Moreover, we pay attention to studies describing increased HM toxicity in the presence of this catechin and the mechanisms through which EGCG may be involved in this effect. Finally, two issues are identified as potential obstacles challenging researchers. These are the low EGCG bioavailability and the safe EGCG dosing to achieve optimal responses without inducing adverse effects. We describe these problems to give a more complete picture of the EGCG potential in the therapy of HM-induced disorders.

## 2. Heavy Metals As Toxicants: Health Risks and Sources for Exposure

In this review, we focused on six HMs i.e., As, Cd, Pb, Hg, Cr(VI), and Ni. We chose these metals taking into consideration the data of ATSDR and EPA, which regard four of these elements (As, Cd, Pb, and Hg) as highly threatening to human health [13], as already mentioned in the background, and the data of IARC, which classify Cd, As, Ni, and Cr(VI) as humans carcinogens [14].

### 2.1. Lead

People can be exposed to lead (Pb) through inhalation of lead-containing particles emitted during metallurgical smelting or stripping leaded paint and through ingestion of Pb-contaminated dust, water (from leaded pipes), and food (from Pb-contaminated containers). The other sources of toxic Pb exposure include unregulated cosmetics and medicines [53]. After absorption, Pb is distributed to mineralized tissues and soft tissues (liver, kidney, lungs, brain, spleen, muscles, and heart). Most Pb is accumulated in bones and teeth from where it can be mobilized in certain conditions such as lactation, pregnancy, broken bones, and kidney diseases thus elevating blood lead levels even after ceased Pb exposure [54]. Lead can induce harmful effects on the central nervous system (CNS) and kidneys as well as the immune, reproductive, and cardiovascular systems [15]. Children, infants in neonatal periods, and the fetus are particularly sensitive to Pb toxicity [55]. The high sensitivity of children to Pb results from the fact that they can absorb significantly higher amounts of Pb from a given source than adults e.g., children can absorb 50% of Pb ingested after a meal, whereas adults absorb 20% [54]. Additionally, children retain more Pb in the body and store more Pb in the brain [56]. The target system of Pb toxicity in children is the developing nervous system, which in turn exerts adverse effects on specific functional domains such as memory, language functions, and attention and executive functioning [56]. For example, it was estimated that as many as 0.5 mln children under 6 years in the USA had blood Pb levels ≥5 μg/dL [57]. Noteworthy, blood Pb concentrations even below 5 μg/dL (50 μg/L) are associated with subtle effects on the intelligence quotient (IQ) in children, and these effects worsen with higher blood lead levels [58]. Currently, the highest risk of Pb poisoning in children is noted in developing countries. As described by Amadi et al. [15], in recent years, many cases of Pb poisoning including deaths have been reported in Nigerian children with sources of exposure from mining, ore processing, agriculture, or use of Pb-containing cosmetic and medicines. In high-income countries such as the USA, the main source of Pb poisoning in children is via the exposure to Pb-contaminated dust and chips from deteriorating Pb indoor paints [59].

### 2.2. Arsenic

Arsenic (As) is a carcinogenic element contaminating groundwater in many parts of the world, especially in South Asian countries. Bangladesh, Pakistan, China, India, Nepal, and Cambodia are substantially affected by As groundwater pollution with As levels often much greater than the WHO arsenic limit of 10 μg/L. The main source of As groundwater pollution in these areas is considered to be in their geology [60]. In addition, the use of arsenical pesticides, inappropriate disposal of arsenical chemicals [61], and burning of high-As coal may also release considerable amounts of As to the environment [32]. In addition to water, rice is the major plant-derived food that significantly contributes to human exposure to As [62]. The most characteristic symptoms of chronic As intoxication are dermal lesions, i.e., pigmentation of the body and keratosis of the palms and soles. Other clinical manifestations include weakness, anemia, neuropathy, liver enlargement, lung disease, and peripheral arterial disease [30]. According to IARC, As and inorganic As are human carcinogens (group 1) causing cancer of the lung, urinary bladder, and skin. Positive associations with kidney, liver, and prostate cancer were found as well [63].

### 2.3. Cadmium

The kidney is the major target organ in Cd toxicity. In addition, Cd can induce bone demineralization directly or indirectly through renal dysfunction. Cd is retained in the kidney and liver with an extremely long biological half-life of 10–30 years, making this metal undoubtedly dangerous in the long term. In a non-smoking general population, Cd exposure is mainly associated with ingestion of cereals and vegetables grown in Cd-polluted soils [64]. In addition, smoking may significantly contribute to Cd intake; smokers were reported to have 4–5 times higher Cd blood levels than non-smokers [65]. According to an EFSA report for the European population, vegetarians, children, smokers, and people living in contaminated areas may exceed the Cd tolerable weekly intake (TWI) (2.5 μg/kg bw) approximately two times [64]. The most severe form of chronic Cd toxicity is the disease called itai-itai reported in Toyama, Japan. The disease was officially recognized in 1968 to be caused by consumption of Cd-polluted water and rice. An intervention program targeted at elimination of Cd pollution was introduced in 1980–2012. Nevertheless, the cases of itai-itai disease were reported over subsequent years after removal of Cd pollution due to the high bioaccumulative features of this metal [66]. Recently, in some Cd-contaminated regions of southern China, the mean grain Cd concentrations have been found to be in the range of 0.33–0.69 mg/kg, among which 56–87% of samples exceeded the Chinese food limit for Cd (0.2 mg/kg) [67]. The authors of this work have estimated that the dietary Cd exposure of farmers who consume locally grown rice is comparable with that of people living in the region of Japan affected by itai-itai disease [67].

### 2.4. Chromium (VI)

Chromium (VI) is a carcinogenic metal whose principally inhalation-related exposure was associated with lung cancer in workers employed in chromate and chromate pigment factories and chromium electroplating [68]. In addition, an epidemiologic study conducted in the industrial Oinofita region in Greece suggested carcinogenic potential of Cr(VI) also in oral ingestion. In this study, an elevated Cr(VI) concentration in drinking water (maximum levels in the range of 41–156 μg/L) was suggested to contribute to a higher risk of cancers of liver, lungs, and genitourinary organs [69]. Cr(VI) from anthropogenic sources is a known aquatic pollutant threatening various fish species [70]. Recently, Cr concentrations in gills of freshwater fishes of the genus *Barbus* (*B. sharpeyi*) from the Tigris River, Baghdad, have been reported to be 2.20 and 2.5 μg/g at two sampling sites. These values significantly exceeded the WHO and FAO maximum permissible limit of HMs in freshwater fishes (0.05 μg/g) [71].

### 2.5. Nickel

People may be exposed to nickel (Ni) compounds in occupational settings where Ni is produced, processed, or used, including mining, smelting and refining, stainless steel production, Ni alloy production, electroplating, and Ni-Cd battery manufacture [72]. Epidemiologic studies found an increased risk of respiratory cancers (nasal and pulmonary) in Ni refinery and smelter workers, and IARC classified Ni compounds as group 1 carcinogens [73]. In addition, Ni is the most common cause of contact allergy in susceptible persons. Ni allergy is usually manifested by eczematous dermatitis within the skin area of direct contact with the metal [74]. In addition to occupational exposure sources, Ni-containing objects like cell phones [75], jewelry [76], and metallic implants [77] were reported as potential, non-occupational sources of Ni allergy.

### 2.6. Mercury

There are three forms of mercury (Hg) in the environment: elemental (or metallic, Hg^0^) Hg, inorganic Hg compounds, and organic Hg compounds. All these forms are toxic [78]. Elemental Hg is a silver-colored heavy metal occurring in its liquid state at room temperature. It is present in such devices as older fever thermometers, fluorescent bulbs, dental amalgams, and jewelry items (e.g., some necklaces from Mexico) [79,80]. Mercury-containing devices are regarded as potential non-occupational sources of metallic Hg exposure, since they release metallic Hg in the form of toxic odorless vapor when broken or heated. Metallic Hg poisoning cases with mild to death-threatening health symptoms were reported upon inhalation exposure to Hg vapors from vacuumed or heated metallic Hg. The poisoning symptoms included acute (e.g., cough, dyspnea, chest pain) and chronic effects (e.g., rash, tremor, and weight loss) (reviewed by Caravati et al. [80]). As reviewed by Park and Zheng [78], inorganic Hg is used as a skin lightening ingredient of cosmetic creams, and cases of Hg poisoning from these products were reported in Africa, Europe, the US, Mexico, Australia, and China. The toxic effects developing after dermal exposure to inorganic Hg included fatigue, irritability, headaches, insomnia, burning sensations, and depression [78]. Organic Hg, i.e., ethylmercury (EtHg) used as a preservative (thimerosal), is still present in vaccines for pregnant women, infants, and children in developing countries. Although vaccines contain a very low dose of this compound, which should not be toxic according to WHO, their safety is questioned by some researchers [81]. Another organic Hg is methylmercury (MeHg), which is contained in contaminated seafood (fish and marine mammals) and rice grown in mercury-polluted areas of China [12]. Methylmercury is an established environmental neurotoxicant ranked as the most toxic form among Hg compounds (MeHg > EtHg > Hg) [82].

## 3. Absorption, Metabolism, and Bioavailability of Epigallocatechin Gallate

EGCG is mainly absorbed intestinally, in the jejunum and the ileum, via passive diffusion, including paracellular and transcelullar diffusions through epithelial cells [40,83]. Following absorption, EGCG is present in plasma mostly in an unchanged free (unconjugated) form [84,85]. From circulation, EGCG can be distributed to other tissues in the body and has been found in the liver, kidney, spleen, lung, and brain [86]. EGCG was also detected in fetuses and placenta of pregnant rats [40]. In the liver and intestine, EGCG is metabolized through methylation to 4′′-O-methyl-EGCG and 4′,4′′-di-O-methyl-EGCG [87]. It can also undergo glucuronidation or sulfation [88]. In addition, nonabsorbed EGCG can also be hydrolyzed by intestinal microflora producing epigallocatechin (EGC) and gallic acid; next, EGC degrades to ring-fission products [39]. A ring-fission metabolite 5-(5′-hydroxyphenyl)-γ-valerolactone in a conjugated form was identified in urine of rats following oral administration of radioactive EGCG [89]. Other ring-fission metabolites, namely 5-(3′,4′,5′-trihydroxyphenyl)-γ-valerolactone, 5-(3′,4′-dihydroxyphenyl)-γ-valerolactone, and 5-(3′,5′-dihydroxyphenyl)-γ-valerolactone, were detected in human urine after ingestion of pure EGCG [90]. In rats, 77% of the [4-^3^H]-EGCG dose was excreted in the bile and only 2% of the dose was found in urine, which shows that bile is the major EGCG excretion route [91].

The poor oral bioavailability of EGCG is largely caused by its poor intestinal stability and absorption. EGCG is chemically very unstable in the intestinal pH conditions [92]. For example, Zou et al. [93] observed that the residual EGCG content in an EGCG solution dropped from 97.9% to 3.4% after 1.5 h incubation in simulated intestinal fluid (weakly alkaline pH). As mentioned above, EGCG is also prone to degradation by intestinal microflora. Additionally, both the poor intestinal transport of EGCG related to passive diffusion and the active efflux of EGCG back into the lumen caused by ATP-dependent efflux proteins (multidrug resistance related proteins MRP1 and MRP2) contribute to the low intestinal absorption of this catechin [92]. Thus, only a small part of ingested EGCG can reach the bloodstream and can be distributed to other tissues. For example, as reported by Nakagava and Miyazawa [94], EGCG concentrations in fasted plasma were 1047 ng/mL in rats and 156 ng/mL in humans, which represented only 0.012% and 0.32% of ingested EGCG, respectively. Another study reported that the mean peak plasma concentration of EGCG in humans was 77.9 ng/mL (0.17 μM) after taking a single oral dose of green tea solid (containing 195 mg of EGCG) dissolved in 200 mL of water (equivalent of two cups of tea) [85]. However, human volunteers taking 500 mg of EGCG as capsules (without food) with 100 mL of water had a maximum plasma EGCG concentration of 824.2 ng/mL (1.8 μM) [95].

EGCG and its metabolites were found to be absorbed in organs and tissues in the body, with the highest concentrations found in the small intestine and colon, as described below. For example, following administration of a single dose of 150 mg/kg to rats, the highest concentrations of free EGCG were revealed in small intestine and colon, reaching the values of 4.75–24.41 nmol/g. Free EGCG was also found in the liver, kidney, spleen, lung, and brain at the levels of 0.1–1 nmol/g [86]. In another study, the authors showed that after administration of EGCG (500 mg/kg bw, orally) to rats, the EGCG concentrations reached the values of 12.3 nmol/mL in the plasma, 48.4 nmol/g in the liver, 0.5 nmol/g in the brain, 565 nmol/g in the small intestine, and 68.6 nmol/g in the colon [96]. Additionally, ring-fission metabolites of microbial EGCG degradation were present in the plasma [39]. The ring-fission metabolites of catechins were shown to contribute largely to the bioavailability of flavan-3-ols in humans [97]. They exhibited cytoprotective activities including antioxidant and anti-inflammatory effects and were therefore suggested to contribute to the EGCG-mediated biological activities [39].

## 4. Mechanistic Considerations of the Protective Effects of EGCG against HM Toxicity

The focus in this review is placed on experimental studies that have tested EGCG in animal and cell culture models as a potential antidote against HM toxicities. Potentially eligible studies were identified by searching Pubmed, Scopus, and additional manual search through references of previous papers. The following search terms were used: [(heavy metals) or (lead) or (arsenic) or (cadmium) or (mercury) or (nickel) or (chromium)] AND [(epigallocatechin gallate) or (EGCG)]. The collected studies and their results are described in Table 2 (animal data) and Table 3 (cell culture studies). Most of them prove the beneficial effects of EGCG in the treatment of HM poisoning. Considering the intrinsic antioxidant properties of EGCG, several mechanisms through which EGCG may alleviate the adverse actions of HMs have been suggested, as described below.

### 4.1. Direct Antioxidant Effects of EGCG via Scavenging Cytotoxic Reactive Species and Metal Chelation

EGCG has the capacity to scavenge free radicals and nonradical reactive species directly. It owes this property to the three vicinal hydroxyl groups on the B ring and on the gallate moiety (D-ring) [121]. The antioxidative mechanism involves H-atom transfer from the active hydroxyl groups to the free radical (Ar-OH + R· → ArO· + RH) [122]. In a pure chemical system, EGCG neutralized superoxide anion and hydrogen peroxide and prevented hydroxyl radical-induced DNA damage [43]. EGCG was also shown to be a peroxynitrite scavenger decreasing the nitration of tyrosine [123] and a scavenger of peroxyl radicals [124], hydroxyl radicals [125], hypochlorite [42], and model free radicals, such as DPPH (1,1-diphenyl-2-picryl-hydrazyl) and ABTS (2,2′-azino-bis-[3-ethylbenzothiazoline-6-sulfonic acid] diammonium salt) [41]. Another free radical scavenging mechanism of EGCG may involve chelation of iron and other metals through EGCG phenolic groups [40]. EGCG was found to bind metals such as Fe(III) [50,125], Cu(II) [126], Cd [113], and Pb [127] to inactive forms, which may contribute to the reduction of the amounts of available free forms of these metals for prooxidant reactions. For example, due to its Fe^3+^ binding capacity, EGCG can possibly decrease the labile Fe^3+^ pool, which prevents Fe^2+^-based Fenton-type reactions [121].

Recently, studies have shown that EGCG (25 and 50 mg/kg bw for 15 days, orally) protected against As-induced oxidative stress and augmentation in genotoxic indices in the liver and kidney of mice, probably owing to its radical scavenging and metal chelating activities [102]. In another study, EGCG (10 mg/kg bw for 30 days through gavage) exhibited a protective effect against As-mediated ROS generation and apoptosis induction in the murine thymus [99]. Similar effects were also reported in cell culture studies in which coincubation with EGCG (5–25 μM for 24 h) decreased intracellular ROS levels and protected from Cr(VI)-mediated apoptosis and DNA damage [116]. Similarly, the EGCG cotreatment (50 μM for 24 h) decreased Pb-induced oxidative stress and apoptotic cell death [120]. EGCG (100 μM for 2 h) was also found to inhibit Cd-induced dysfunction of mitochondria (from rat brain) through reduction of mitochondrial lipid peroxidation [113]. In addition, the same authors performed spectroscopic analysis of Cd and EGCG interactions, demonstrating the formation of Cd and EGCG complexes in a 1:1 ratio, which was also suggested to contribute to the EGCG-mediated protection of mitochondria. The Cd-EGCG complex was formed at pH 8.3, and the stability of the complex significantly declined at pH 7.6 [113]. However, the HM chelating properties of EGCG and their role in its radical scavenging activity was questioned by other authors. For instance, An et al. [114] noticed that, although EGCG (20 μM for 3 h) protected cultured liver cells from Cd-induced apoptosis through ROS scavenging, there was no EGCG and Cd binding in their experimental conditions i.e., at neutral pH of the solution. No Cd-EGCG complex formation at the same pH value as that used by An et al. [114] was also reported by Yu et al. [112]. The results of these studies showed that the experimental conditions can significantly influence the ability of EGCG to chelate Cd^2+^.

As mentioned earlier in this review, some HMs (e.g., Cd or Pb) have a high tendency to accumulate in soft tissues (e.g., kidney or brain) and bones, thus posing a long-term health risk. Certain data in literature support the notion that, probably due to its metal chelating capacity, EGCG may be beneficial in promoting mobilization and excretion of HMs from tissues simultaneously with improvement of oxidative stress markers, as described below. For example, Han et al. [104] have noted that oral application of EGCG (50 mg/kg bw for 30 days, intragastric) to rats lowered the As content in the liver, decreased oxidative stress, and protected the liver from histopathological changes caused by As. Similarly, Yu et al. [99] revealed that EGCG (10 mg/kg bw for 30 days, intragastric) administration to As-exposed mice decreased As levels in the thymus and spleen and attenuated the prooxidant and proapoptotic effects of As on the thymus. Another study demonstrated the preventive effects of EGCG (200 mg/kg bw once a week for 6 months orally) on bioaccumulation of HMs from a multi-heavy metal mixture in rats [128]. The results of this study showed reduced Hg accumulation in the serum, heart, lung, brain, and liver, a lower Cd concentration in the liver, spleen, and kidney, and reduced Cr and Ni accumulation in the spleen and serum [128]. The researchers suggested that, by promotion of the excretion of HMs from the body, EGCG partly alleviated the joint toxicity of these metals in the serum and liver. In contrast to the above-described results, a study conducted by Yin et al. [107] found that cotreatment with EGCG (25 and 50 mg/kg for 10 days i.p.) of Pb-exposed rat pups increased Pb levels in the blood. It was suggested that, via complexing Pb^2+^, EGCG increased its lipophilicity and gastrointestinal Pb absorption. Nevertheless, the EGCG administration in this study was beneficial in protecting the rat brain from Pb-induced oxidative stress [107].

### 4.2. Regulation of the Nrf2 Antioxidant Pathway

Nrf2 (nuclear factor [erythroid-derived 2]-like 2) is mostly known as a transcription factor activating antioxidant genes, including genes of heme oxygenase-1 (HO-1), NAD(P)H:quinone oxidoreductase-1 (NQO1), and superoxide dismutase (SOD1 and SOD2) and genes of enzymes essential for GSH and NADPH synthesis. In addition, Nrf2 stimulates xenobiotic detoxification genes that encode enzymes involved in e.g., glutathione and sulfonate conjugation, glucoronidation, and metallothionein expression. Nrf2 activation is also involved in suppression of inflammatory responses, increased mitochondrial biogenesis and improvement of mitochondrial functions [129]. Since most of the Nrf2-induced responses mentioned above can contribute to HM detoxification, the activation of the Nrf2 protein seems to play an important protective role against adverse HM outcomes. Indeed, as shown by Toyama et al. [130,131], Nrf2-deficient mice (Nrf2^−/−^) and primary hepatocytes derived from Nrf2-deficient mice were highly sensitive to MeHg toxicity, in comparison with wild-type mice (Nrf2^+/+^) or primary hepatocytes from Nrf2^+/+^ mice.

The activation of Nrf2 at a low to moderate level of oxidative stress is recognized as an initial adaptive response aiming to suppress oxidative stress and maintain cellular homeostasis. However, during high oxidative stress, the Nrf2 pathway is inhibited and more cell destructive inflammatory responses (e.g., NF-κB activation) are triggered [132]. Thus, the inhibition of Nrf2 in response to HM exposure was indicated as a mechanism contributing to the toxicity and adverse health effects of heavy metals. For instance, treatment with individual HMs (Cd, Pb, Hg, and Cr) has recently been shown to impair the Nrf2 signaling pathway in THP-1 monocytes, which was connected with a decline in lung function, lower plasma GST activity and GSH level in HM-exposed chronic obstructive pulmonary disease patients [133].

EGCG is classified as one of the natural products that can activate the Nrf2 protein [134]. The mechanism of EGCG-mediated Nrf2 activation has been associated with activation of the PI3K (phosphatidylinositol 3-kinase)/Akt pathway and ERK1/2 (extracellular signal-regulated protein kinase 1/2) signaling [135]. It can therefore be suspected that EGCG can prevent HM toxicity via stimulation of Nrf2 antioxidant pathways. Accordingly, EGCG (10 μg/mL for 48 h) was demonstrated to activate Skn-1, i.e., the Nrf2 ortholog in worms, thereby protecting *C. elegans* from MeHg toxicity, as shown by Chen et al. [117]. The researchers observed reduced oxidative stress and alleviation of neurotoxic effects, which was confirmed by improved locomotion behaviors and lower numbers of damaged neurons. In another study, EGCG treatment (40 mg/kg for 14 days) upregulated Nrf2 expression in the kidney of mice which enhanced antioxidant system and protected from As-induced toxicity [101]. Contrarily, reduced nuclear accumulation of Nrf2 was demonstrated in response to EGCG (50 μM for 24 h) treatment of As-exposed human keratinocytes [111]. This observation was associated with potentiation of As-mediated prooxidant and genotoxic effects, which may suggest excessive oxidative stress that probably suppressed the activation of Nrf2. So far, no further research studies that analyze the association between Nrf2 stimulation and EGCG effects on HMs have been reported.

### 4.3. Regulation of Inflammatory Responses

Inflammation is a protective mechanism in the body against tissue injury. It involves release of inflammatory mediators, including cytokines and chemokines, and reactive oxygen/nitrogen species (ROS/RNS) as well as attraction of leukocytes to the damaged site in order to eliminate the cause of the injury. The inflammation process is a beneficial response since it allows elimination of tissue damage. However, due to the deregulation or chronic exposure to adverse factors, the ongoing state of inflammation can cause excessive tissue damage and contribute to the development of various diseases, e.g., allergy, asthma, atherosclerosis, autoimmune diseases, and cancer [44,136]. Inflammatory reactions often involve activation of fundamental proinflammatory transcription factors such as the nuclear factor kappaB (NF-κB) and activator protein 1 (AP-1). Once activated, NF-κB and AP-1 move from the cytoplasm to the nucleus and promote the expression of proinflammatory genes, including those coding for cytokines [137,138]. Inflammatory responses induced by HMs frequently play a significant role in the progression of HM-related diseases.

EGCG (10 μM for 24 h) was shown to suppress inflammatory responses, such as activation of NF-κB and AP-1 in mouse epidermal cells, thereby reducing the cytotoxicity of Ni nanoparticles (Ni NPs) [118]. The authors also demonstrated that EGCG inhibited the upregulation of protein expression of mitogen activated protein kinases (MAPK), namely p-JNK, p-ERK1/2, and p-p38. Since the upregulation of MAPK is very likely to cause activation of NF-κB and AP-1, EGCG-mediated downregulation of MAPK was suggested as a mechanism involved in the EGCG-mediated inhibition of NF-κB and AP-1 activity, which led to attenuation of Ni NPs-mediated cell toxicity [118]. Yu et al. [99] demonstrated that EGCG (10 mg/kg for 30 days by gavage) inhibited As-induced inflammation in mice, as evidenced by a decreased level of nitric oxide (NO) and suppressed release of proinflammatory cytokines (IL-6, TNFα, IL-1β) in serum [99]. In addition, Chen et al. [106] reported that, by targeting the TGF-β1/Smad3 signaling pathway, EGCG protected against Cd-induced renal injury and fibrosis in rats. These beneficial effects of EGCG were also accompanied by modulation of renal microRNA levels i.e., decreasing the level of microRNA-21 (miR-21) and miR-192 and increasing the levels of miR-29a/b/c in the Cd-treated rats [106].

### 4.4. Regulation of Mitochondrial Functions

Emerging evidence suggests that mitochondria may play a major role in the beneficial effects of EGCG. As reviewed by Oliveira et al. [139], EGCG may affect diverse mitochondrial functions related to mitochondrial biogenesis, bioenergetics (e.g., ATP synthesis), alterations in cell cycle, and the mitochondrial-dependent apoptotic pathway. A clear participation of mitochondria in the biological actions of EGCG was evidenced by Schroeder et al. [140]. The researchers demonstrated that as much as 90–95% of EGCG accumulated in the mitochondria of rat cerebellar granule neurons (CGNs) and protected these cells only from those toxic stimuli that induced apoptosis through mitochondrial oxidative stress. In this experimental model, EGCG (10 and 20 μM, for 24 h) protected CGNs from apoptosis induced by such mitochondrial oxidative stressors as HA14-1 (Bcl-2 inhibitor), *tert*-butylhydroperoxide (generator of H_2_O_2_), and SIN-1 (generator of peroxinitrite). On the other hand, EGCG (5, 10, or 20 μM for 24 h) did not protect CGNs from proapoptotic insults that are independent of oxidative stress such as proteasome inhibitor (MG132) or trophic factor withdrawal. The authors suggested that accumulation of EGCG in mitochondria, free radical scavenging, and transition metal chelating capabilities were critical in the EGCG-mediated protection of mitochondria in CGNs. Simultaneously, they suggested that the protective effects of EGCG on mitochondria may be cell type specific or stimulus specific and other mechanisms of EGCG may be induced in other cell types [140].

Some studies conducted with various experimental models were focused on the EGCG effects on mitochondria following HM exposure. Some of these studies have already been mentioned in this review in the context of free radical scavenging and metal chelating capabilities of EGCG. An example is the report by Abib et al. [113], who showed the protective activity of EGCG (100 μM for 2 h) against Cd-induced impairment of mitochondria present in mitochondrial-enriched fractions from rat brain. Another study reported by An et al. [114] demonstrated that, through its radical scavenging properties, EGCG (20 μM for 3 h) was effective against collapse of the mitochondrial membrane potential and mitochondrial apoptotic pathway caused by Cd in normal human liver cells. In an in vivo experimental model, EGCG administration (20 mg/kg for 40 days i.p.) to As-exposed mice reduced oxidative stress and restored mitochondrial membrane potential in spermatozoa, thereby contributing to improvement of sperm quality [103]. Some mechanisms through which EGCG may exert its protective effects on mitochondria during HM exposure can be derived from the study conducted by Pan et al. [141]. These authors used a mitochondrial fraction isolated from the kidney of mice exposed to platinum-containing cisplatin. They demonstrated that EGCG (100 mg/kg i.p. for 2 days) ameliorated mitochondrial oxidative/nitrative stress and improved the activities of mitochondrial respiratory enzyme complex activities (NADH dehydrogenase activity, succinate dehydrogenase activity, and cytochrome oxidase activity) and the activities of mitochondrial antioxidant enzymes i.e., manganese superoxide dismutase (MnSOD) and glutathione peroxidase (GPx). This led to reduction of cisplatin-mediated renal injury [141].

Overall, EGCG was found to elicit protective effects on mitochondria during HM exposure in different experimental models through reducing oxidative stress, preserving mitochondrial membrane potential, and enhancing mitochondrial antioxidant and respiratory functions. It is plausible that, through its property of accumulation in mitochondria, EGCG may act as an antioxidant within these organelles, protecting them from HM-induced injury.

**Figure 2:**
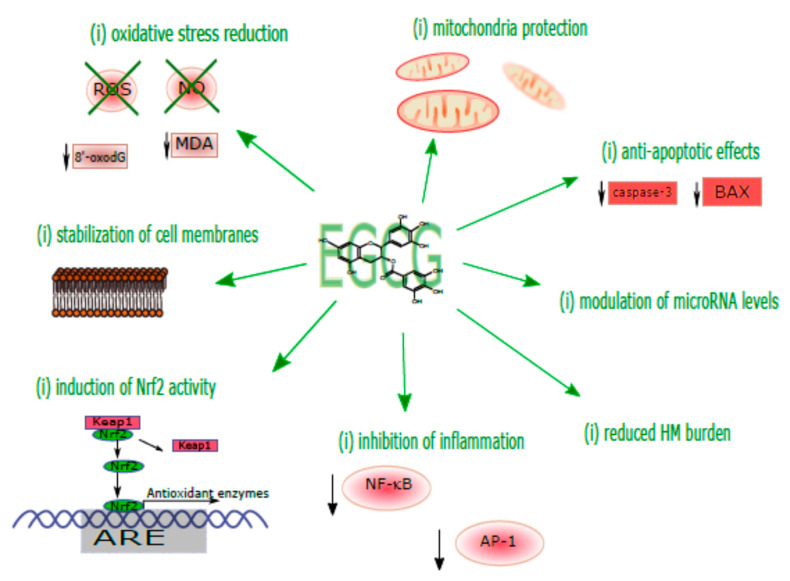
Major protective mechanisms observed in EGCG effects on HM toxicity in experimental studies. AP-1, activator protein 1; ARE, antioxidant response element; Keap1, Kelch-like ECH-associated protein 1; MDA, malondialdehyde; NF-κB, nuclear factor-kB; NO, nitric oxide; Nrf2, nuclear factor erythroid 2-related factor 2; 8′-oxodG, 8-oxo-2′-deoxyguanosine; ROS, reactive oxygen species; ↓, decreased.

The mechanisms of the EGCG protective action relevant to the toxicological pathology of HMs are summarized in Figure 2.

## 5. Toxic Effects Triggered by EGCG during HM Exposure

Although the majority of studies presented in Table 2 and Table 3 proved the beneficial effects of EGCG on HM toxicity, some studies especially from cell culture models have found that EGCG may also exert converse actions and potentiate the harmful outcomes of HM exposure. For instance, EGCG (50 μM for 24 h) significantly increased the prooxidant and genotoxic effects of arsenite in HaCaT cells [111]. The exact mechanism was not examined by the authors, but it may be explained by the oxidation of EGCG in the cell culture conditions resulting in the formation of ROS and toxic EGCG metabolites. For example, one study reported that, in in vitro conditions in Tris-HCl buffer (at pH 7.2), EGCG underwent degradation to form several oxidation products, including EGCG quinone, EGCG dimer quinone, and EGCG dimers [142]. The authors proposed a mechanism of chain reactions of EGCG autooxidation, during which EGCG was oxidized by molecular oxygen to produce ROS and several unstable metabolites with EGCG quinone as the key intermediate. The ROS and EGCG degradation products were suggested to provoke various cellular effects. It is uncertain however whether such reactions can occur in vivo [142]. In addition, through its ability to reduce iron ions from Fe^3+^ to Fe^2+^, high doses of EGCG may accelerate Fenton reactions and generation of hydroxyl radicals [43]. Nakazato et al. [109] reported that EGCG (10 μM for 24 h) significantly enhanced As_2_O_3_-induced apoptosis in human malignant B-cell lines, including myeloma cells (RPMI8226) and Burkitt’s lymphoma cells (HS-sultan). Moreover, the combination of EGCG (10 μM for 3 h) and As_2_O_3_ caused depletion of the intracellular GSH level and enhancement of intracellular ROS levels (O_2_^−^ and H_2_O_2_) in HS-sultan and myeloma cells (IM9 cells). The depletion of GSH and higher levels of ROS were suggested as the main mechanism responsible for the increased apoptosis induction observed during the combined EGCG and As_2_O_3_ treatment [109]. In another study, Kim et al. [110] demonstrated the mechanism by which EGCG made primary-cultured bovine aortic endothelial cells (BEAC) more prone to arsenite-induced toxicity. In this study, EGCG and arsenite combined at nontoxic doses (20 μM) activated the JNK pathway, which decreased the activity of catalase leading to increased ROS production, triggering Bax translocation into the mitochondria, and activating proapoptotic enzymes, which consequently resulted in induction of apoptotic cell death [110]. Other mechanisms that may also contribute to toxicity of high doses of EGCG include high affinity of EGCG for lipid bilayers of cell membranes [143,144] as well as EGCG-induced uncoupling of mitochondrial oxidative phosphorylation and damage to the outer mitochondrial membrane [145].

Very recently, Bondad et al. [115] found that low dose of EGCG (1.5 μM) significantly increased the adverse effects of Cd on cell viability and membrane integrity in neural PC12 cells. The authors could not explain the mechanism of the EGCG effects on Cd toxicity, however, EGCG may lack antioxidant activity in certain cell lines, irrespective of its dose, as demonstrated by Elbling et al. [146]. These researchers showed that EGCG at concentration ranges between 0.01 and 50 μM was not able to inhibit H_2_O_2_-induced ROS generation and genotoxicity in human promyelocytic leukemic HL60 cells. In this study, EGCG administered at higher but physiologically relevant concentrations (1 μM and higher) enhanced the H_2_O_2_ genotoxic effects and, starting from 10 μM, increased H_2_O_2_-dependent oxidative stress induction [146].

Overall, EGCG may potentiate the toxic effects of HMs, and these were observed mostly in cell culture studies. The adverse effects of EGCG were observed both in cancerous and noncancerous cell lines. In addition, there is a wide range of EGCG doses that can enhance the harmful action of HMs in vitro spanning from 1.5 μM (as observed in the study conducted by Bondad [115]) to 50 μM (as observed by Sarkar et al. [111]). As mentioned above, many different mechanisms may be involved in the adverse actions of EGCG during HM exposure. Nonetheless, the low stability of EGCG in solutions and its tendency to autooxidation resulting in ROS formation was suggested as an artifact that may significantly influence the EGCG effects in in vitro conditions [147,148]. For example, 1 mM EGCG was found to induce generation of 90 and 141 μM H_2_O_2_ in Dulbecco’s Modified Eagle’s Medium (DMEM) and DMEM/F12 culture medium, respectively. In this study, transition metals present in cell culture media (e.g., Fe and Cu) were suggested to catalyze EGCG autooxidation and contribute to H_2_O_2_ production [147]. Therefore, it is important to verify the ability of tested concentrations of EGCG to induce H_2_O_2_ production in examined cell culture conditions.

## 6. Potential Obstacles in the Use of EGCG in HM Intoxication Treatment

The low bioavailability of EGCG is generally regarded as the main impediment in the use of EGCG for the therapy of various diseases, since it hinders the achievement of therapeutic concentration levels of EGCG in the target tissues. Different factors diminish EGCG bioavailability, in particular its poor intestinal stability and low absorption through the intestinal gut wall. In addition, the intestinal and liver-mediated extensive metabolism of EGCG (methylation, glucuronidation, and sulfation) and microbial-mediated degradation in the colon also influence the utilization of EGCG [83,92]. There have also been reports on inter-individual variations of EGCG plasma concentrations after oral administration of GT extracts (150 mg EGCG) twice daily (for 5 days) among 84 healthy subjects. On the 5th day of oral administration, the plasma area under the curve (AUC) of EGCG ranged from 360.8 to 1576.5 h * μg/L and the elimination half-lives were in the range of 1.8–3.8 h, as measured by their 5th to 95th percentiles [149]. The authors suggested that inherent genetic variations in genes coding for drug transporters, namely multidrug resistance-associated protein (MRP) 2 and organic anion-transporting polypeptide (OATP) 1B1, could partly influence the variability in catechin plasma concentrations [149]. Different strategies have been suggested to increase EGCG bioavailability. The use of nanocarriers as EGCG delivery systems is being intensively investigated, as it contributes to improvement of intestinal stability and absorption of EGCG. For example, casein micelles were suggested as protective nanocarriers for EGCG in food products [150]. Nanoliposome encapsulation of EGCG effectively improved EGCG stability in simulated intestinal fluid and slowed down the degradation rate of in vitro antioxidant activity of EGCG [93]. EGCG-loaded chitosan-tripolyphosphate nanoparticles improved oral absorption of EGCG in mice, as evidenced by the increased accumulation of EGCG in the stomach and jejunum and the increased plasma concentration of EGCG [151]. Other techniques proposed to increase EGCG bioavailability include molecular modification of phenolic hydroxyl groups of EGCG (reviewed by Dai et al. [92]) and coadministration of EGCG with other food ingredients such as sucrose and ascorbic acid [152].

Furthermore, it is worth indicating that although the oral bioavailability of EGCG and other catechins is low, studies suggest that the concentrations of EGCG in certain conditions such as fasting or repeated dosing (administration) can reach toxic values at which EGCG can induce adverse health effects [153]. Therefore, another problem to solve is to establish safe EGCG dose levels allowing efficacious treatment of HM toxicity without the risk of adverse side effects. Concerns over the safe EGCG dosing during the treatment of various diseases have already been raised by some authors. For example, via comparison of different animal studies, Wang et al. [154] concluded that some of the efficacious (or protective) doses of EGCG and toxic doses of this polyphenol are very close or even overlap. For example, intraperitoneal doses of EGCG (50–75 mg/kg for 1–56 days) that were shown to be protective against brain or liver damage in mice were close to those which induced hepatotoxicity (100 mg/kg, single i.p. injection) in the same animal model. With regard to HM treatment, animal data showed that the oral doses of EGCG protecting against HMs were in the range of 10–50 mg/kg (for 1–30 days) in mice and 50–200 mg/kg (for 30–112 days) in rats. For comparison, Table 4 summarizes some literature data on the toxic effects of EGCG and EGCG-containing preparations administered through the oral or i.p. route in rodents. Thus, it was evidenced that, for example, oral administration of GT extract containing 242 mg EGCG/kg (for 14 weeks) induced nasal toxicity in rats [155]. This dose was close to a dose of EGCG (200 mg/kg for 16 week, orally) that proved to be protective against Cd renal toxicity in rats [106]. With regard to EGCG treatment via the i.p. route, there is one study in which the EGCG dose (80 mg/kg/d for 49 days) protecting against Pb-induced damage to rat testes [108] was not far from an EGCG dose (108 mg/kg, a single i.p. treatment), that was found to induce toxic effects in the liver of rats [156]. However, some EGCG doses protecting against HM toxicity administered through the oral or intraperitoneal route in mice and rats were much lower than doses reported to cause toxic effects in rodent models. For example, in a study of As-mediated cancer, diet with 0.05% EGCG was sufficient to protect mice against As carcinogenic effects. This EGCG dose was far from that in a 1% EGCG diet reported to induce proinflammatory effects in mice [157].

Overall, it is known that antioxidants administered at high doses can have adverse effects, and the data presented above indicate that there may be a narrow boundary between the protective and toxic doses of EGCG. This certainly makes EGCG a challenge for its potential therapeutic application in HM toxicity treatments. Enhancement of the bioavailability of EGCG and reduction of its toxicity through food nanotechnology, structural modification, or beneficial interactions may be a way of overcoming the adverse responses.

## 7. Conclusions and Future Directions

In recent years, many studies have demonstrated the effects of EGCG on HM-induced toxicity in in vitro and in vivo experimental conditions. In general, the in vivo data showed that EGCG can help ameliorate HM toxicity via its ROS scavenging activity, promotion of HM excretion, induction of Nrf2 expression, anti-inflammatory effects, and protection of mitochondria. This protective activity of EGCG was observed in various organs and tissues, including the liver, testis, kidney, and neuronal tissue. Indeed, in spite of its low bioavailability, pharmacokinetic data show that EGCG can be distributed to different internal organ/tissues and cross the blood–brain barrier, thus reaching tissues affected by HM intoxication. The results of the assessment of EGCG effects on HM toxicity from in vitro models are ambiguous. On the one hand, most of them support the in vivo data on the EGCG protective effects and mechanisms induced against HMs. On the other hand, some in vitro results showed potentiation of HM toxicity in the presence of EGCG at similar EGCG concentrations (1.5–20 μM), as its beneficial effects were observed. This may be related to the differences in the susceptibility of particular cell types or differences in cell culture conditions, including artifactual generation of hydrogen peroxide or other reactive molecules as a result of autooxidation of EGCG in cell culture media. Noteworthy, the analysis of some in vivo data suggests that indeed the range between nontoxic and toxic EGCG doses may be narrow, which may hamper the EGCG use in the treatment of HM intoxication. This issue certainly needs further study.

In conclusion, future research is warranted in this field of science. The following aspects have been found as worth to be considered:

1. Estimation of optimal EGCG dose ranges which are both safe and effective in the treatment of HM toxicity;
2. Investigation of the indirect mechanisms by which EGCG can modulate HM toxicity, including mitochondrial functions and Nrf2 activity, using different mammalian cells or tissues that are particularly prone to HM intoxication such as the lung, brain, liver, or kidney;
3. Analysis and comparison of the efficacy of native EGCG and nanoEGCG in HM toxicity treatment;
4. Verification of the possible synergistic effect between EGCG and chelation agents on HM toxicity;
5. Investigation of the effects of microbial ring-fission metabolites of EGCG and their contribution to EGCG effects on HM toxicity.

## Figures and Tables

**Table 1:** Potential non-occupational exposures to heavy metals (HMs).

HMs	Exposure Source	Adverse Effects on Human Health	Ref.
Ni, V	Fine particulate matter in the urban environment	Possible contribution to respiratory symptoms in children (in New York City)	[17]
Cd	Cigarette smoke	Increased risk of cardiovascular diseases (in Sweden)	[18]
Cd	Rice	Increased risk of kidney diseases (in Japan)	[19]
Cd, Al	Shellfish	Allergic eczema	[20]
As	Fish	Possible contribution to nonmelanoma skin cancer (in Singapore)	[21]
As	Highland barley	Increased probability of cancer risk (in western Tibet)	[22]
Ti	Titanium-based dental and orthopedic implants	Allergic eczema	[23]
V	Titanium-based orthopedic implants	Systemic dermatitis	[24]
Pb	Ayurvedic medicines	Abdominal pain	[25]
Pb	Coffee	Possible contribution to disease burden in heavy coffee drinkers	[26]
Al	Cosmetics (antiperspirants)	Possible contribution to breast cancer	[27]

**Table 2:** Summary of animal studies evaluating EGCG effects on HM-induced toxicity.

EGCG Dose	HM Dose	Duration of Treatment	Model	Relevant EGCG Interferences	Suggested Mechanism/s of Action	Ref.
0.05% in powder chow	DMA(V): 400 ppm, drinking water following lung tumor initiator 4NQO injection	DMA(V) and EGCG cotreatment for 25 weeks	Mice	↓ incidence of lung tumors ↓ 8-oxodG level in lungs	ROS scavenging	[98]
10 mg/kg i.g.	NaAsO_2_ 10 mg/kg, i.g.	NaAsO_2_ and EGCG cotreatment for 30 days	Mice	↓ NO level and IL-1β, IL-6, and TNFα release in serum; ↓ CD8 (cytotoxic) T cell and ↑ CD4 (helper) cell frequency; ↑ CD3-positive T cell and CD19-positive B cell frequency; ↓ As content in the thymus and spleen; In the thymus: ↓ ROS, ↓ caspase-3 activity, ↑ MMP, ↓ apoptotic and necrotic cell number	ROS scavenging/anti-inflammatory effects/metal chelation	[99]
10 mg/kg, i.g.	CrO_3_ 20 mg/kg i.p.	Single EGCG treatment followed by CrO_3_ injection	Mice	Peripheral blood: ↓ micronucleated polychromatic erythrocytes, ↓ cell viability, ↑ apoptotic and necrotic cell number	Proapoptotic effects	[100]
40 mg/kg i.g.	NaAsO_2_ 20 mg/kg i.g.	NaAsO_2_ and EGCG cotreatment for 14 days	Mice	↑ sperm motility; ↓ As content in the liver and kidney; ↓ LPO level in the kidney and lung; ↓ PCC level in the lung and brain; ↑ GSH level in the liver, kidney, testis, and brain; ↑ SOD activity in the testis and brain; ↑ GST activity in the liver, kidney, lung, testis, and brain; ↑ BChE in the brain; ↑ Nrf2 expression in kidney; ↓ histopathological changes in the brain; no effect on DNA damage in blood cells	ROS scavenging/metal chelation/increased Nrf2 signaling	[101]
25 and 50 mg/kg orally	NaAsO_2_ 1.5 mg/kg i.p.	(1) EGCG pretreatment for 15 days followed by 10 days of As treatment (2) As treatment for 10 days followed by EGCG post-treatment for 15 days	Mice	In the liver and kidney: ↓ LPO, ↑ GSH level and ↑ activity of antioxidant enzymes (CAT, SOD, GST, GR), ↓ DNA damage; Bone marrow cells: ↓ chromosomal aberrations and formation of micronuclei; Blood lymphocytes: ↓ comet tailing; ↓ ROS;	ROS scavenging/metal chelation	[102]
20 mg/kg i.p.	As 200 ppm (drinking water)	As and EGCG cotreatment for 40 days	Mice	Epididymal sperm: ↑ concentration, ↑ kinematic attributes, ↑ membrane integrity, ↑ MMP; In the testis: ↓ LPO, ↑ GSH level and CAT activity; ↓ histopathological changes	ROS scavenging/stabilization of mitochondria	[103]
50 mg/kg i.g.	NaAsO_2_ 5 mg/kg i.g.	NaAsO_2_ and EGCG co-treatment for 30 days	Rats	In the liver: ↓ AST, ALT, ALP, and LDH activity, ↑ SOD and CAT activity, ↑ GSH level, ↓ MDA and ROS level, ↓ As content, ↓ histopathological changes	ROS scavenging/metal chelation	[104]
50 mg/kg i.g.	NaAsO_2_ 5 mg/kg i.g.	NaAsO_2_ and EGCG co-treatment for 30 days	Rats	Heart tissue: improved morphology and ultrastructure, ↓ As content, ↓ apoptotic cell number, ↑ integrity of plasma membrane, ↑ SOD, CAT and GPx activity, ↓ MDA level, ↓ intracellular Ca^2+^ concentration	ROS scavenging/maintenance of intracellular Ca^2+^ levels	[105]
100 and 200 mg/kg i.g.	CdCl_2_ 250 mg/L in (drinking water)	CdCl_2_ and EGCG cotreatment for 16 weeks	Rats	↓ blood urea nitrogen and serum creatinine; in the kidneys: improved morphology, ↓ collagen deposition and fibrosis, ↓ TGF-β1 and p-Smad3 level, ↑ GSH level, ↑ SOD and GPx activity, ↓ MDA and NO level, ↓ miR-21 and miR-192 level and ↑ miR-29a/b/c level	ROS scavenging/anti-inflammatory effects/modulation of microRNA levels	[106]
10, 25, and 50 mg/kg i.p.	Pb acetate 1090 ppm (drinking water)	Pb acetate treatment from PND1-20 (via mother’s milk) and PND21–23 (via drinking water) EGCG cotreatment from PND14–23	Rats (pups)	↑ Pb in blood; in the hippocampus: ↑ long-term potentiation amplitude in the CA1 area, ↑ GSH level and SOD activity, ↓ MDA level	ROS scavenging/metal chelation	[107]
80 mg/kg i.p.	Pb acetate 50 mg/L (drinking water)	Pb acetate and EGCG cotreatment for 49 days	Rats	↑ sperm motility, ↑ relative weight of testis and seminal vesicles, ↑ serum testosterone and 17β-estradiol level, in the testis: ↑ *cyp19* (aromatase P450) gene expression, ↑ SOD, CAT, and GPx activity, ↓ MDA levels, ↑ testicular architecture and semen picture	ROS scavenging/increased *cyp19* gene expression	[108]

ALP, alkaline phosphatase; ALT, alanine aminotransferase; AST, aspartate transaminase, BChE, butyrylcholinesterase; CAT, catalase; DMA(V), dimethylarsinic acid; GSH, reduced glutathione; GR, glutathione reductase; GPx, glutathione peroxidase; GST, glutathione S-transferase; IL, interleukin; LDH: lactic dehydrogenase, LPO, lipid peroxidation; MDA, malonyldialdehyde; miR, microRNA; NaAsO_2_: sodium arsenite; NO, nitric oxide; Nrf2, nuclear factor erythroid 2-related factor 2; 4NQO, 4-nitroquinolin 1-oxide; 8′-oxodG: 8-oxo-2′-deoxyguanosine; PCC, protein carbonyl content; ROS, reactive oxygen species; SOD, superoxide dismutase; TGF-β1, transforming growth factor-β1; TNFα, tumor necrosis factor-αc; ↑, increased; ↓, decreased.

**Table 3:** Summary of studies evaluating EGCG effects on HM-induced toxicity in cell culture models.

EGCG Concentration	HM Dose	Duration of Treatment	Cell Type	Effects of EGCG on the Toxicity of HMs	Suggested Mechanism/s of EGCG Action	Ref.
10 μM	As_2_O_3_ 2 μM	As_2_O_3_ and EGCG coincubation for 3 or 24 h	Myeloma cells (RPMI 8226, IM9), Burkitt’s lymphoma cells (HS-sultan)	↓ cell viability; ↑ apoptotic cells; ↑ intracellular ROS; ↓ GSH level; ↓ Bcl-2, Mcl-1, and procaspase-3 protein level	Increased ROS production/decreased GSH levels/proapoptotic effects	[109]
20 μM	NaAsO_2_ 20 μM	NaAsO_2_ and EGCG coincubation for 3–24 h	Primary bovine aortic endothelial cells (BAEC)	↓ cell viability; ↑ number of apoptotic cells; ↑ caspase 3, 8, and 9 activity; ↑ bax translocation into mitochondria; ↑ ROS and MDA level; ↓ CAT activity; ↑ level of phosphorylated JNK (p-JNK)	JNK activation/increased ROS production/proapoptotic effects	[110]
50 μM	NaAsO_2_ 50 μM	NaAsO_2_ and EGCG coincubation for 24 h	Normal human keratinocytes HaCaT cells	↑ ROS and MDA level; ↑ 8-OHdG content; ↑ DNA damage (comet assay); ↓ nuclear and ↑ cytosolic expression of Nrf2; ↑ nuclear expression of Keap1; ↑ protein expression of HO-1 and γ-GCSC; ↓ SOD, NQO1 and GST activity	Increased ROS production/modulation of Nrf2 signaling pathway	[111]
30 and 150 μM	CdCl_2_ 30 and 50 μM	CdCl_2_ and EGCG coincubation for 24 h	Human prostate cancer cell line PC-3	↓ cell viability; no complex of EGCG with Cd was formed at pH 7.0	Modulation of Ca^2+^ and Zn^2+^ absorption by cells	[112]
100 μM	CdCl_2_ 200 μM	CdCl_2_ and EGCG coincubation for 2 h	Mitochondrial-enriched fractions from rat brain	↑ mitochondrial viability; ↓ mitochondrial LPO; no effects on nonprotein thiol levels; formation of Cd:EGCG complex in a 1:1 ratio at pH 8.3	ROS scavenging/stabilization of mitochondria/metal chelation	[113]
20 μM	CdCl_2_ 60 μM	CdCl_2_ incubation for 21 h followed by coincubation with EGCG for 3 h	Normal human liver cells HL-7702	↑ cell viability; ↓ apoptosis rate; ↓ ROS and MDA levels; ↑ MMP; ↓ caspase 3 activity; EGCG and Cd did not form complexes with each other at neutral pH (pH 7.2)	ROS scavenging/stabilization of mitochondria	[114]
1.5 μM	CdCl_2_ 5 μM	1 h EGCG pretreatment followed by 48 h CdCl_2_ exposure	Rat pheochromocytoma cell line PC12	↓ cell viability; ↓ cell membrane integrity	Increased ROS production/cell membrane disruption	[115]
5, 10 and 25 μM 25–200 μM	K_2_CrO_4_ 10 μM K_2_CrO_4_ 50 μM	K_2_CrO_4_ and EGCG coincubation for 24 h K_2_CrO_4_ and EGCG coincubation for 24 h	Human normal bronchial epithelial BEAS-2B cells Epstein-Barr virus-transformed human Burkitt’s lymphoma EBV-BL	↑ cell viability; ↓ apoptotic cells; ↓ ROS; ↓ mRNA expression of cell death-related genes (*GADD45A*, *PPP1R15A*, *EGR1*); ↑ mRNA expression of genes involved in cell defense (*SMUG1*, *XRCC4* and *ERCC4*) ↓ DNA-protein cross-links	ROS scavenging/modulation of gene expression	[116]
10 mg/mL	CH_3_HgCl (MeHg) 2.5, 5 and 10 μM	48 h EGCG pretreatment followed by 24 h MeHg exposure	*Caenorhabditis elegans*	EGCG alone (without MeHg): ↑ *skn-1* (Nrf2 ortholog) mRNA levels; ↑ *gst-4* gene induction, ↑ total antioxidant ability EGCG treatment followed by MeHg: ↑ LC50 of MeHg (from 26.1 to 30.7 M); ↓ LPO; ↑ locomotion activity; ↓ degeneration of DA and γ-GABA neurons;	Increased Nrf2 signaling pathway	[117]
10 μM	Ni NPs 2.5–10 μg/cm^2^	Ni NPs and EGCG coincubation for 24 h	Mouse epidermal cells JB6	↑ cell viability and morphology; ↑ G0/G1 phase arrest and ↓ G2/M phase arrest; ↓ apoptotic cells; ↓ intracellular ROS generation; ↓ AP-1 and NF-B activation; ↓ protein expression of p-ERK1/2, p-JNK, and p-p38	ROS scavenging/anti-inflammatory effects/modulation of the MAPK signaling pathway	[118]
5, 10, 15 μM	Pb^2+^ 100 μM	Pb^2+^ and EGCG coincubation for 24 h	Human hepatocellular carcinoma cell line HepG2	↑ cell viability; ↓ LPO; ↑ cell membrane fluidity	ROS scavenging/metal chelation/stabilization of cell membranes	[119]
50 μM	Pb acetate 5 μM	Pb acetate and EGCG coincubation for 24 h	SH-SY5Y human neuroblastoma cells	↓ apoptosis rate; ↓ ROS levels; ↓ caspase 3 activity; ↓ bax/bcl2 ratio	ROS scavenging/antiapoptotic effects	[120]
50 μM	Pb acetate 20 μM	Pb acetate and EGCG coincubation for 24 h	Primary hippocampal neurons	↑ cell viability; ↓ ROS levels; ↑ MMP	ROS scavenging/stabilization of mitochondria	[107]

ALP, alkaline phosphatase; ALT, alanine aminotransferase; AP-1, activator protein 1; AST, aspartate aminotransferase, DA, dopamine; γ-GABA, γ-aminobutyric acid; γ-GCSC, γ-glutamylcysteine synthetase heavy subunit; GSH, reduced glutathione; GST, glutathione S transferase; HO-1, heme oxygenase-1; Keap1, Kelch ECH associating protein 1; LDH, lactate dehydrogenase; LPO, lipid peroxidation; MDA, malonyldialdehyde; MeHg, methylmercury; MMP, mitochondrial membrane potential; NF-κB, nuclear factor-κB; Nrf2, nuclear factor erythroid 2-related factor 2; NQO-1, NAD(P)H:quinone oxidoreductase 1; 8-OHdG, 8-hydroxy-2′-deoxyguanosine; p-ERK1/2, phosphorylated extracellular signal-regulated kinase; p-JNK, phosphorylated c-Jun N-terminal kinase; ROS, reactive oxygen species, SOD, superoxide dismutase; ↑, increased; ↓, decreased.

**Table 4:** Selected literature data on the toxic effects of EGCG and EGCG-containing green tea extracts or preparations in rodents.

EGCG Dose	Route of Administration	Duration	Animals	Toxic Effects	References
EGCG 100 mg/kg	i.p.	4 d	Swiss albino mice 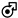 (diabetic)	Death (60% animals); ↑ serum cystatin C and NGAL (markers of kidney damage) In the kidney: ↑ NADPH oxidase, ↓ TAC, GSH, Nrf2, HO-1, and HSP 90, ↑ NF-κB and TNF-α, ↑ histopathological changes	[158]
EGCG 55 mg/kg	i.p.	5 d	Kunming mice 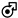	↓ body weight; in the serum: ↑ ALT, AST (markers of liver damage), ↑ 4-HNE, IL-2, IL-6 and IL-10	[159]
EGCG 50 mg/kg	i.p.	3 d	DO mice 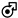	Mild liver injury (0.55–9.94% liver necrosis) in 49% animals. Severe liver injury (10–86.8% liver necrosis) in 16% animals	[160]
GT extract 62.5, 125, 250, 500, and 1000 mg/kg containing 30.3–484 mg/kg of EGCG (48.4%)	i.g.	14 weeks (5 days per week)	B6C3F1/N mice 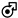 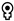	Death, 6 of 10 ( 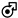 ) and 4 of 10 ( 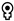 ) at 1000 mg GT/kg (484 mg EGCG/kg) In the nose, starting from 250 mg GT/kg (121 mg EGCG/kg, 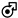 ) and 500 mg GT/kg (242 mg EGCG/kg, 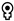 ): nerve atrophy, olfactory epithelium atrophy, olfactory epithelium metaplasia; Reproductive toxicity at 500 mg GT/kg (242 mg EGCG/kg); In the liver: centrilobular necrosis ( 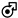 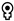 ) and karyomegaly ( 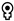 ) at 1000 GT mg/kg (484 mg EGCG/kg)	[155]
EGCG 1500 mg/kg 750 mg/kg	i.g.	Single dose 2–7 d	CF-1 mice 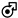	85% in mortality (1500 mg/kg) and 75% in mortality (750 mg/kg); In the plasma: ↑ ALT, MCP-1 and IL-6; In the liver: ↑ apoptosis and necrosis of hepatocytes, ↑ 4-HNE, ↑ metallothionein I/II	[161]
GT extract 200 mg/kg containing 108 mg/kg of EGCG (54%)	i.p.	Single dose	SPF rats 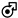 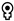	12% in mortality ( 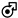 ), 50% in mortality ( 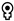 ); In the serum: ↑ AST, ALT and MDA ( 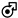 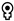 ); In the liver: ↑ apoptosis and necrosis of hepatocytes, ↑ MDA- and TG-positive hepatocytes, ↑ inflammatory reactions ( 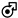 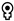 )	[156]
GT extract 62.5, 125, 250, 500, and 1000 mg/kg containing 30.3–484 mg/kg of EGCG (48.4%)	i.g.	14 weeks (5 days per week)	F344/NTac rats 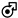 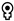	Reproductive toxicity ( 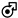 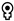 ) at 1000 mg GT/kg (484 mg EGCG/kg); In the liver ( 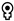 ): hepatocyte necrosis, bile duct hyperplasia, oval cell hyperplasia and mitosis at 1000 mg GT/kg (484 mg EGCG/kg); In the nose starting from 500 mg GT/kg (242 mg EGCG/kg): nerve atrophy and olfactory epithelium metaplasia ( 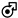 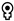 ), inflammation ( 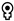 )	[155]

ALT, alanine aminotransferase, AST, aspartate aminotransferase; DO, diversity outbred; GSH, reduced glutathione; GT, green tea; 4-HNE, 4-hydroxynonenal; HO-1, hemeoxygenase-1; HSP90, heat shock protein 90; IL, interleukin; MCP-1, monocyte chemoattractant protein 1; NF-kB, nuclear factor kappa-B; NGAL, neutrophil gelatinase-associated lipocalin; Nrf2, nuclear factor erythroid 2-related factor 2; TAC, total antioxidant capacity; TG, thymidine glycol; TNF-α, tumor necrosis factor-α.
